# Impact of Interleukin-17 Receptor A Gene Variants on Asthma Susceptibility and Clinical Manifestations in Children and Adolescents

**DOI:** 10.3390/children11060657

**Published:** 2024-05-28

**Authors:** Shymaa Ahmed Maher, Nouran B. AbdAllah, Essam Al Ageeli, Eman Riad, Shahad W. Kattan, Sherouk Abdelaal, Wagdy Abdelfatah, Gehan A. Ibrahim, Eman A. Toraih, Ghada A. Awadalla, Manal S. Fawzy, Ahmed Ibrahim

**Affiliations:** 1 Department of Medical Biochemistry and Molecular Biology, Faculty of Medicine, Suez Canal University, Ismailia 41522, Egypt; shimaa.maher@med.suez.edu.eg; 2 Center of Excellence in Molecular and Cellular Medicine (CEMCM), Faculty of Medicine, Suez Canal University, Ismailia 41522, Egypt; 3 Department of Pediatrics, Faculty of Medicine, Suez Canal University, Ismailia 41522, Egypt; nouran_bayoumi@med.suez.edu.eg (N.B.A.); shrouq_rahman@med.suez.edu.eg (S.A.); ahmed_ibrahim@med.suez.edu.eg (A.I.); 4 Department of Basic Medical Sciences, Faculty of Medicine, Jazan University, Jazan 45141, Saudi Arabia; ealageeli@jazanu.edu.sa; 5 Department of Chest Diseases and Tuberculosis, Faculty of Medicine, Suez Canal University, Ismailia 41522, Egypt; emanriad@med.suez.edu.eg (E.R.); wagdyabdelfatah@med.suez.edu.eg (W.A.); 6 Department of Medical Laboratory, College of Applied Medical Sciences, Taibah University, Yanbu 46423, Saudi Arabia; skattan@taibahu.edu.sa; 7 Department of Clinical Pathology, Faculty of Medicine, Suez Canal University, Ismailia 41522, Egypt; gehan_salem@med.suez.edu.eg; 8 Department of Surgery, School of Medicine, Tulane University, New Orleans, LA 70112, USA; etoraih@tulane.edu; 9 Medical Genetics Unit, Department of Histology and Cell Biology, Suez Canal University, Ismailia 41522, Egypt; 10 Biochemistry Department, Animal Health Research Institute, Mansoura Branch, Giza 12618, Egypt; ghadaawadalla2017@gmail.com; 11 Department of Biochemistry, Faculty of Medicine, Northern Border University, Arar P.O. Box 1321, Saudi Arabia

**Keywords:** asthma, interleukin 17RA, genetic association, polymorphisms, real-time PCR

## Abstract

Several single nucleotide polymorphisms (SNPs) in multiple interleukin receptor genes could be associated with asthma risk and/or phenotype. Interleukin-17 (IL-17) has been implicated in tissue inflammation and autoimmune diseases. As no previous studies have uncovered the potential role of *IL17 receptor A* (*RA*) gene variants in asthma risk, we aimed to explore the association of four *IL17RA* SNPs (i.e., rs4819554A/G, rs879577C/T, rs41323645G/A, and rs4819555C/T) with asthma susceptibility/phenotype in our region. TaqMan allelic discrimination analysis was used to genotype 192 individuals. We found that the rs4819554 G/G genotype significantly reduced disease risk in the codominant (OR = 0.15, 95%CI = 0.05–0.45, *p* < 0.001), dominant (OR = 0.49, 95%CI = 0.26–0.93, *p* = 0.028), and recessive (OR = 0.18, 95%CI = 0.07–0.52, *p* < 0.001) models. Similarly, rs879577 showed reduced disease risk associated with the T allele across all genetic models. However, the A allele of rs41323645 was associated with increased disease risk in all models. The G/A and A/A genotypes have higher ORs of 2.47 (95%CI = 1.19–5.14) and 3.86 (95%CI = 1.62–9.18), respectively. Similar trends are observed in the dominant 2.89 (95%CI = 1.47–5.68, *p* = 0.002) and recessive 2.34 (95%CI = 1.10–4.98, *p* = 0.025) models. For the rs4819555 variant, although there was no significant association identified under any models, carriers of the rs4819554*A demonstrated an association with a positive family history of asthma (71.4% in carriers vs. 27% in non-carriers; *p* = 0.025) and the use of relievers for >2 weeks (52.2% of carriers vs. 28.8% of non-carriers; *p* = 0.047). Meanwhile, the rs4819555*C carriers displayed a significant divergence in the asthma phenotype, specifically atopic asthma (83.3% vs. 61.1%; *p* = 0.007), showed a higher prevalence of chest tightness (88.9% vs. 61.5%; *p* = 0.029), and were more likely to report comorbidities (57.7% vs. 16.7%, *p* = 0.003). The most frequent haplotype in the asthma group was ACAC, with a frequency of 22.87% vs. 1.36% in the controls (*p* < 0.001). In conclusion, the studied *IL17RA* variants could be essential in asthma susceptibility and phenotype in children and adolescents.

## 1. Introduction

Asthma is a prevalent chronic respiratory illness in pediatrics, characterized by mucus hypersecretion, persistent airway inflammation, and bronchial hyper-responsiveness, leading to the narrowing of the bronchi and increased resistance to airflow, often reversible naturally or with treatment [1]. It is prevalent worldwide, especially in developed countries where the prevalence is increasing to epidemic proportions. According to the statistical survey of the “Global Asthma Network’s Phase 1”, the prevalence of asthma symptoms was 9.1%, 11.0%, and 6.6% for children, adolescents, and adults, respectively [2].

Asthma is a multifaceted disease influenced by various factors, including genetics. Discovering genes associated with asthma, understanding genetic variations, and examining cellular signaling pathways could be crucial for predicting disease outcomes and developing targeted treatments [3]. It is considered a classic T-cell-mediated condition driven by various cytokines orchestrating the inflammatory response. Predominantly, these include the Th2-secreted cytokines such as interleukin-4 (IL-4), IL-5, and IL-13. Additionally, IL-17 plays a notable role in the development and severity of asthma [4].

**Figure 1:**
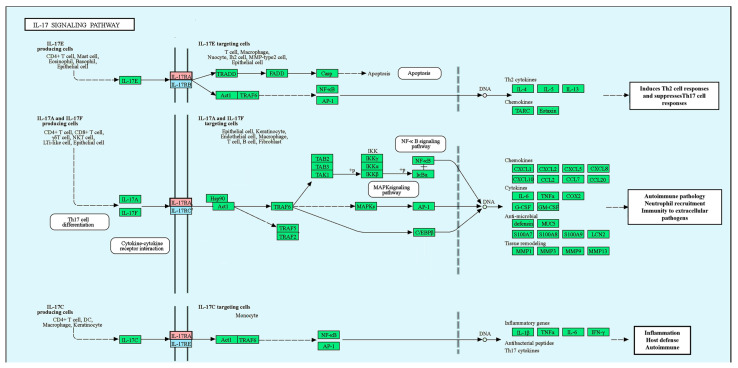
Interleukin-17 signaling pathway (Kyoto Encyclopedia of Genes and Genomes (KEGG) ID: Human (Homo sapiens), hsa04657). The IL-17 cytokine family (IL-17A-F) plays a role in acute and chronic inflammation. IL-17A, predominantly secreted by T helper 17 (TH17) cells, is essential for protecting against external pathogens and is implicated in autoimmune inflammation. IL-17F primarily protects mucosal surfaces, while IL-17E promotes Th2 immune responses. IL-17C shares similar functions with IL-17A. The IL-17 family communicates via specific receptors, such as IL17RA, which several family members use. Activation of downstream signaling pathways, including NF-kappa B, mitogen-activated protein kinases (MAPKs), and CCAAT-enhancer-binding proteins (C/EBPs), results in the production of antimicrobial peptides and the expression of cytokines and chemokines. Adopted from (https://www.genome.jp/pathway/hsa04657+23765) (last acceded 7 July 2023) [9,10].

The IL-17 cytokine family, which significantly influences both acute and chronic immune responses [5], is composed of six members: IL-17A, B, C, D, E, and F. Within this family, IL-17A and IL-17F are linked explicitly to T helper type 17 (Th17) cells [6]. These cytokines signal through five receptors named IL-17 receptors (IL-17R) A–E. Among these, IL-17A primarily signals via the IL-17R complex, which includes the IL-17RA and IL-17RC subunits, with IL-17RA displaying a higher binding affinity [7,8] (Figure 1) [9,10].

Interleukin (IL)-17A, the primary cytokine produced by T helper 17 (Th17) cells, plays a crucial role in defending host mucosal surfaces [11] and acts as a significant inflammatory mediator in various autoimmune diseases, such as rheumatoid arthritis (RA) [12,13]. The IL-17A/IL-17RA signaling pathway has been associated with the development of several acute and chronic respiratory conditions, including asthma [14,15,16,17,18,19,20]. Research has shown that IL-17A promotes the growth and survival of airway smooth muscle cells in both asthmatic and non-asthmatic individuals through IL-17RA activation [21]. Additionally, IL-17A can trigger the production of inflammatory cytokines by human bronchial fibroblasts, including IL-6, IL-8, and IL-11, potentially leading to further inflammation and tissue remodeling [16].

In patients with asthma, there is an upregulation of IL-17 mRNA and proteins found within the lungs, sputum, and bronchoalveolar lavage fluids [4,22]. Research has shown that respiratory infections caused by viruses, bacteria, and fungi can impact the Th17/IL-17A pathway, consequently exacerbating both the onset and progression of asthma [23,24].

Previous studies have indicated that various single nucleotide polymorphisms (SNPs) in several interleukin and interleukin receptor genes, including IL-4 and IL-4R [25,26,27,28], IL-9 [29], IL-10 [30], IL-5 and IL5R [31], IL13 [32], IL-33 and its receptor IL-1R-like 1 [33,34], IL-18 [35], IL-13R [36], and IL-23R [37], may be linked to an increased risk of developing asthma.

Although several SNPs related to IL-17A have been linked to susceptibility of a variety of immune-mediated/inflammatory disorders [38,39,40,41,42,43,44], including asthma [45,46,47,48,49], with potential modification of related gene expression and Th17 cells activities [40,50], no previous studies have explored the potential role of *IL17RA* gene variants in asthma susceptibility, in particular in our population. In this sense, the current study was designed to investigate for the first time the association between four SNPs (i.e., rs4819554, rs879577, rs41323645, and rs4819555) in *IL-17RA* and asthma susceptibility/phenotype in our region. These SNPs were chosen according to the criteria outlined in the Methods section.

## 2. Materials and Methods

### 2.1. Population Characteristics

Pediatric patients, ranging in age from 6 to 15 years, diagnosed with asthma were included from the “Pediatrics, Allergy, and Immunology, as well as Chest Diseases and Tuberculosis” clinics at the Suez Canal University Hospital in Ismailia, Egypt. The research was carried out among two distinct groups, with 96 unrelated patients diagnosed with bronchial asthma, comprising the asthma group and 96 age-similar healthy individuals (i.e., the control group). Diagnoses were formulated following the Global Initiative for Asthma (GINA) regulations. Notable factors like co-existing health conditions, the extent of control over asthma symptoms over the previous month, disease seriousness, treatment level and responsiveness, and consistency of therapy adherence were evaluated by the treating physicians based on GINA’s guidelines [51]. To ensure accuracy and standardization in line with GINA recommendations, we implemented a secondary review process. Prior to the inclusion of participants in the study, the initial evaluations made by the treating physicians underwent an additional layer of verification by a separate team of respiratory specialists. This team was not involved in the initial clinical care of the patients, providing an independent confirmation of the asthma classification.

Chest X-rays were taken to rule out alternate causes of airflow restrictions. Asthma patients with chronic comorbidities, those who exhibited signs of respiratory infection in the previous three weeks, those treated with systemic steroids in the past fortnight, or those non-adherent to therapy were not included in the study for several reasons: (1) impact on test results: steroid therapy can suppress inflammation and modify airway responsiveness, potentially altering the results of bronchial challenge tests. Excluding patients recently receiving steroid therapy helps ensure the test accurately reflects their underlying respiratory condition. (2) Consistency of results: patients not adherent to their medication regimen may have variable airway inflammation and responsiveness, making it challenging to interpret the results of bronchial challenge tests reliably. Excluding non-adherent patients helps maintain consistency in the study population and improves the validity of the findings. (3) Safety concerns: in some cases, patients not adherent to their medication regimen may have poorly controlled asthma or other respiratory conditions, putting them at higher risk of adverse events during bronchial challenge testing. Excluding these patients helps mitigate potential safety concerns and ensures the ethical conduct of the study.

Control participants were children accompanying their siblings to the Pediatrics Clinic, with no precedent of atopic disorders, history of medications, or any health problems, including chronic respiratory diseases. The “body mass index (BMI)” was determined by dividing the weight of the individual (in kilograms) by their height (in meters) raised to the power of two [52]. Pubertal development was evaluated using the Tanner stages of growth [53].

Adherence to the “Declaration of Helsinki” outlines was ensured. The research gained approval from the “Medical Research Ethics Committee” at the Faculty of Medicine, Suez Canal University, Ismailia, Egypt, and parents or guardians of the study participants provided their written consent.

### 2.2. Pulmonary Function and Methacholine Challenge Tests

Baseline pulmonary function tests were conducted using the ExpAir spirometry system from Medisoft (Viasys Healthcare, Conshohocken, PA, USA), following the guidelines of the American Thoracic Society (ATS) and European Respiratory Society (ERS) [54]. The parameters measured included “peak expiratory flow rate (PEFR)”, “forced vital capacity (FVC)”, and the “forced expiratory volume in one second (FEV1)”. Participants were administered 400 µg of salbutamol (known commercially as Ventolin by GlaxoSmithKline) using a metered-dose inhaler and a spacer. Forced spirometry was repeated 15 min after administering the bronchodilator salbutamol. The response to salbutamol was calculated using the following formula: “[BDRBASE = ((post-salbutamol FEV1 − pre-salbutamol FEV1)/pre-salbutamol FEV1) × 100]” [55].

Airway hyperresponsiveness was evaluated using a methacholine challenge test, following the clinical practice guidelines [56]. The amount of methacholine required to induce a 20% reduction in FEV1 was determined and recorded in mg/mL. A PC20 value of less than 8 mg/mL indicated positive airway hyperresponsiveness [57].

### 2.3. Laboratory Investigations

Venous blood samples were collected in EDTA and plain tubes. The EDTA tubes were used for absolute peripheral blood eosinophil count using the “Coulter counting method (Beckman Coulter Ltd., Brea, CA, USA)” and confirmed by manual microscopic differential count. Eosinophil counts exceeding 400 cells/mm^3^ indicated absolute eosinophilia [58]. Total serum IgE levels were measured from the plain tubes using an enzyme-linked immunosorbent assay (ELISA) with the “Accu-Bind^®^ ELISA kit (Monobind Inc., Lake Forest, CA, USA)”. Total IgE levels of ≥100 IU/mL were classified as an elevated level.

### 2.4. Genomic DNA Extraction and IL17RA Variants Allelic Discrimination Analysis

Genomic DNA isolation was performed from peripheral blood leukocytes utilizing the “Wizard^®^ Genomic DNA Purification Kit (Promega Co., Madison, WI, USA)”. Its quantity was validated through absorptance measurement at 260/280 nm using a “NanoDrop-1000 spectrophotometer (NanoDrop Tech., Wilmington, NC, USA)” to ensure all the included samples in the subsequent analyses have a ratio of 1.8 to 2.0. The DNA samples that did not meet this criterion were either re-purified or excluded from further analysis. Then, the extracted DNA samples were maintained at −20 °C [59].

Based on searching the *IL17RA* gene variants in the “National Center of Biotechnology Information Single nucleotide polymorphism database (dbSNP; www.ncbi.nlm.nih.gov)”, (accessed on 20 September 2022) for a minor allele frequency (MAF) ≥ 0.1, documented relationships to other similar diseases, and lack of previous studies exploring the association of these variants with asthma, in particular in our population, the authors selected the studied SNPs. The genotyping of these SNPs was executed using “real-time polymerase chain reaction (PCR)” allelic discrimination TaqMan assays (cat# 4351379). The assay IDs and the context sequence of each variant with the VIC/FAM-identified polymorphisms are presented in Table 1.

The PCR was executed in a 25 µL reaction volume that includes 20 ng of the extracted DNA, 12.5 µL of TaqMan Universal PCR Master Mix (comprising AmpErase UNG, AmpliTaq Gold enzyme, dNTPs, and reaction buffer), and 1.25 µL of TaqMan SNP genotyping assay (Table 1). All plates had negative controls to exclude cross-contamination. PCR amplification was conducted using the “StepOne™ real-time PCR system (Applied Biosystems, Waltham, MA, USA)”, based on two initial holds (50 °C for 2 min and 95 °C for 10 min) followed by a 40-cycle two-step PCR (95 °C denaturation for 15 s and annealing/extension 60 °C for 1 min) [60]. A randomly chosen 10% of the samples were re-genotyped across different runs to preclude false genotype calls, maintaining a 100% concordance rate. Post-PCR data analysis was carried out using “SDS software (v1.3.1., Applied Biosystems)”.

### 2.5. Statistical Analysis

Statistical analysis was conducted using SPSS version 27.0 (IBM Corp., Armonk, NY, USA). Study power was figured out employing G*Power software (version 3.0.10), revealing an anticipated power of 91% given a total sample size of 192, a medium effect size of 0.3, and an error probability (α) of 0.05. The calculation of allele/genotype and carriage rate frequencies of *IL17RA* adopted the method delineated previously [29]. The “Hardy–Weinberg equilibrium (HWE)” was calculated for each studied variant. Computed odds ratios (OR) with a 95%CI (confidence interval) across varied genetic association models were accomplished through logistics regression models [30]. The potential confounding effects were adjusted using binary logistic regression analysis. The relationship between *IL17RA* SNPs and clinic-laboratory features was assessed using two-sided statistical tests, including the “Chi-square test, t-test, Mann–Whitney U test, or Kruskal–Walli’s test”. A significance level of *p* < 0.05 (two-tailed) was considered statistically significant to determine associations.

### 2.6. In Silico Analysis

The molecular structure of the *IL17RA* gene/protein was determined by searching several available online tools such as the “GeneCards (https://www.genecards.org/), the National Center of Biotechnology Institute (https://www.ncbi.nlm.nih.gov/gene/23765), the Ensembl genome browser 110 (https://asia.ensembl.org/Homo_sapiens/Transcript/), the Protter tool for visualization of proteoforms and interactive integration of annotated/predicted sequence features of the protein (https://wlab.ethz.ch/protter/)” [61], the “UniProt protein database for the resource of protein sequence and functional information (https://www.uniprot.org/)”, “the subcellular/molecular localization of the expressed protein of interest (https://compartments.jensenlab.org/)”, and the “known/predicted protein–protein interactions network generation (https://string-db.org/)” (all previous databases were last accessed on 20 July 2023).

The functional effect of the studied SNPs was checked using the following bioinformatics tools: “MutationTaster (https://www.mutationtaster.org/, accessed on 20 July 2023)” [62]; “SIFT (Sorting Intolerant from Tolerant, https://sift.bii.a-star.edu.sg/, accessed on 20 July 2023)”, which depends on sequence homology/physical properties of amino acids to predict the effect of missense variants on protein function [63]; PANTHER, that provides “comprehensive information about the evolution of protein-coding gene families depending on protein phylogeny, function and genetic variation impacting that function” [64]; Varsome, which generates recommended pathogenicity based on the data available from multiple genomic databases [65]; “I-Mutant v2.0 predictor of protein Stability changes upon base substitution (https://folding.biofold.org/cgi-bin/i-mutant2.0.cgi, accessed on 20 July 2023)” [66]; and “HaploReg v4.2 to predict the impact of variants on regulatory motifs, and the expression from expression quantitative trait loci (eQTL) studies” [67].

## 3. Results

### 3.1. Participants Characteristics

The study consisted of 96 controls and 96 asthma patients, with an average age of around 9.7 years for both groups (*p* = 0.76). The sex distribution was balanced, with females accounting for 58.3% and 54.2% in the control and asthma groups, respectively (*p* = 0.66). A higher proportion of rural residents was observed in the asthma group (66.7% vs. 53.1%, *p* = 0.08). Notably, a higher percentage of individuals in the asthma group reported a family history of asthma (30.2% vs. 7.3%, *p* < 0.001). BMI percentiles were similar across both groups, and pubertal status and Tanner stages were comparable, indicating similar physical development levels (*p* = 0.88 and *p* = 0.65, respectively) (Table 2).

### 3.2. Association of IL17RA Polymorphisms with Disease Risk

Table 3 presents the relationship between *IL17RA* polymorphisms and disease risk across four genetic models—codominant, dominant, recessive, and over-dominant—for four different polymorphisms (rs4819554, rs879577, rs41323645, and rs4819555). The distribution of genotypes in the control group followed the HWE (*p*-values = 0.42, 1.0, 0.27, and 0.82), respectively.

The G/G genotype of rs4819554 significantly reduced disease risk in the codominant model (OR = 0.15, 95% = 0.05–0.45, *p* < 0.001) compared to the reference A/A genotype. This protective effect of the G allele was also observed in dominant (OR = 0.49, 95%CI = 0.26–0.93, *p* = 0.028) and recessive (OR = 0.18, 95%CI = 0.07–0.52, *p* < 0.001) models. Similarly, rs879577 showed a reduction in disease risk associated with the T allele, but it was consistent across all genetic models. In the codominant model, the T/T genotype (OR = 0.13, 95%CI = 0.05–0.36, *p* < 0.001) was associated with a lower disease risk than the reference C/C genotype. The dominant model showed a similar trend (OR = 0.19, 95%CI = 0.10–0.37, *p* < 0.001). The recessive model indicated a lower disease risk for the T/T genotype (OR = 0.28, 95%CI = 0.11–0.70, *p* = 0.004) compared to C/C-C/T genotypes. The A allele of rs41323645 was associated with increased disease risk in all models. The G/A and A/A genotypes had higher ORs of 2.47 (95%CI = 1.19–5.14) and 3.86 (95%CI = 1.62–9.18), respectively. Similar trends were observed in the dominant 2.89 (95%CI = 1.47–5.68, *p* = 0.002) and recessive 2.34 (95%CI = 1.10–4.98, *p* = 0.025) models, indicating an increased disease risk associated with the A allele. For rs4819555, no significant associations were identified in any models.

Table 4 reports the carriage rates of alleles, meaning the “proportion of individuals carrying at least one copy of the specific allele in question”. The alleles of four different polymorphisms (rs4819554, rs879577, rs4819554, and rs4819555) are compared between the control/patient subgroups. For rs4819554, carriers of the A variant were associated with higher disease risk (OR = 4.01, *p* = 0.002), while those of the G allele appeared protected (OR = 0.45, *p* = 0.012). Similarly, the rs879577*C allele was associated with a higher disease risk (OR = 3.87, *p* = 0.002), and the T allele was associated with a lower disease risk (OR = 0.22, *p* < 0.001). For rs41323645, the G allele appeared protective (OR = 0.41, *p* = 0.016), while the A allele was associated with higher disease risk (OR = 2.47, *p* = 0.006). No significant associations were observed for rs4819555 (*p* = 0.32 and *p* = 0.24, respectively).

Subgroup analysis was performed based on puberty status under a dominant model, where the presence of one or more copies of the minor allele is considered. The genotype distributions between patients and controls for four SNPs (rs4819554, rs879577, rs41323645, and rs4819555) were compared for both prepubertal and pubertal groups (Table 5). For rs4819554, in the prepuberty group, having one or more G alleles (A/G or G/G genotypes) was associated with a reduced risk of disease (OR = 0.49, 95%CI: 0.26–0.93) compared to the A/A genotype. This association, however, was not significant in the pubertal group (OR = 0.70, 95%CI: 0.27–1.77). For rs879577, individuals with one or more T allele (C/T or T/T genotypes) had a significantly lower risk of disease in both prepubertal (OR = 0.11, 95%CI: 0.04–0.30) and pubertal (OR = 0.33, 95%CI: 0.13–0.87) groups, relative to those with the C/C genotype. For rs41323645, individuals with one or more A allele (G/A or A/A genotypes) had a higher risk of disease in the prepubertal group (OR = 3.35, 95%CI: 1.30–8.62). This trend was also observed in the pubertal group, but the association was insignificant (OR = 2.47, 95%CI: 0.95–6.43). For rs4819555, no significant associations were detected in either the prepubertal or pubertal groups. However, at least one T allele (C/T or T/T genotypes) was associated with a trend towards higher risk compared to the C/C genotype. Overall, there was no significant interaction between these SNPs and puberty status (*p* > 0.05), suggesting that puberty does not significantly modify the effect of these SNPs on disease risk.

### 3.3. Haplotype Analysis and Disease Risk

Haplotype frequency analysis was performed to identify potential combinations of alleles across the SNPs that might be associated with asthma risk. The most frequent haplotype in the asthma group was ACAC, with a frequency of 22.87%, significantly higher than in the control group (1.36%, *p* < 0.001). This indicates that the ACAC haplotype could be linked to an increased risk of asthma (Table 6). Other haplotypes, including ACGC, ATGC, and GCAC, were also significantly associated with asthma, albeit with lower frequencies than ACAC. Notably, the ACGC haplotype was associated with a reduced risk of asthma (OR = 0.00, 95%CI: 0.00–0.11, *p* = 0.002). The ATGC/GCAC haplotypes exhibited comparable trends. These haplotypes were less frequently observed in the asthma group than in the control group, and their presence was linked to a notably reduced likelihood of developing asthma (OR = 0.00, *p* < 0.001 for ATGC; OR = 0.00, *p* = 0.002 for GCAC). The haplotypes ACGT, ATGT, ACAT, GTAC, GCAT, GTAT, and GTGT were present at different frequencies in asthma and control groups and were significantly associated with lower odds of having asthma (*p* < 0.05). In contrast, haplotype GCGC, GTGC, and ATAC showed no significant association with risk of asthma (*p* > 0.05).

### 3.4. Association of Gene Variants with Disease Severity

In exploring gene variants’ association with disease severity in pediatric asthma, distinct correlations were noted among the carriers of specific *IL17RA* gene variants. While many patient characteristics and disease outcomes exhibited no significant differences between carriers and non-carriers, several exceptions were identified (Table 7).

Notably, carriers of the rs4819554*A allele demonstrated a significant association with a positive family history of asthma, contrasting 71.4% in carriers to 27% in non-carriers (*p* = 0.025). Similarly, rs41323645*A carriers showed a significant correlation with the use of relievers for more than two weeks, with 52.2% of carriers as opposed to 28.8% of non-carriers exhibiting this usage pattern (*p* = 0.047).

Carriers of the rs4819555*C allele displayed a significant divergence in the asthma phenotype, specifically atopic asthma, identified in 83.3% of carriers compared to 61.1% of non-carriers (*p* = 0.007). The same group showed a higher prevalence of chest tightness (88.9% in carriers versus 61.5% in non-carriers, *p* = 0.029). Furthermore, carriers of the rs4819555*C allele were significantly more likely to report comorbidities (57.7% versus 16.7%, *p* = 0.003), with allergic rhinitis and eczema being more common in this group (44.9% and 38.5% respectively, compared to 16.7% in non-carriers, *p* = 0.033 for allergic rhinitis and *p* = 0.001 for eczema). Lastly, a significant difference in eosinophil count was observed between rs4819555*C carriers and non-carriers, with a median count of 50 (IQR: 20–207.5) × 10^6^/L in carriers compared to 230 (IQR: 86–509.8) × 10^6^/L in non-carriers (*p* = 0.009).

Supplementary Table S1 delineates the relationship between the carriage of *IL17RA* alleles and various allergies or aggravating factors contributing to asthma. Notably, emotional stress triggers were significantly associated with the rs41323645*A carriers (*p* = 0.047). Aside from this, our analysis indicates that the studied gene variants do not significantly affect asthma development in the context of these risk factors.

### 3.5. Association of Gene Variants with Therapeutic Response

As depicted in Table 8 on the carriage of risk alleles, the total number of patients varied for each risk allele, with 162 carrying rs4819554*A, 159 carrying rs879577*C, 127 carrying rs41323645*A, and 162 carrying rs4819555*C. The table compares the management of airway hyperresponsiveness (AHR) and therapy levels for each group. Across the spectrum of normal to severe AHR and therapy levels from Step 1 to Step 5, there were no significant differences in frequencies between carriers and non-carriers of the risk alleles, indicated by *p*-values above 0.05. The therapeutic response was assessed using several parameters, including FVC1, Pre- and Post-FEV1, PEFR1, “provocative concentration causing a 20% drop in FEV1 (PC20)”, and “bronchodilator response (BDRBASE)”. Notably, carriers of rs4819554*A marginally significantly lower values for Post FEV1 (% predicted) and PEFR1 (% predicted) than non-carriers, with *p*-values of 0.045 and 0.046, respectively.

### 3.6. Association of IL17RA Haplotypes with Asthma Severity and Therapeutic Response

Table 9 details a comparison of demographic characteristics, disease severity and control, management strategies, and therapeutic responses between two groups differentiated by the presence or absence of the risk haplotype block (rs4819554*A/rs879577*C/rs41323645*A). The last SNP rs4819555 (C/T) was excluded, as it did not show a significant association. This haplotype block is henceforth referred to as ACA (C/T). The total sample comprised 96 participants, 36 without the risk haplotype and 60 with the risk haplotype. The analysis demonstrated insignificant differences (*p*-value > 0.05) in these characteristics between the two groups.

### 3.7. Molecular Features of IL17RA and In Silico Analysis of the Impact of the Studied IL17RA Variants

**Figure 2:**
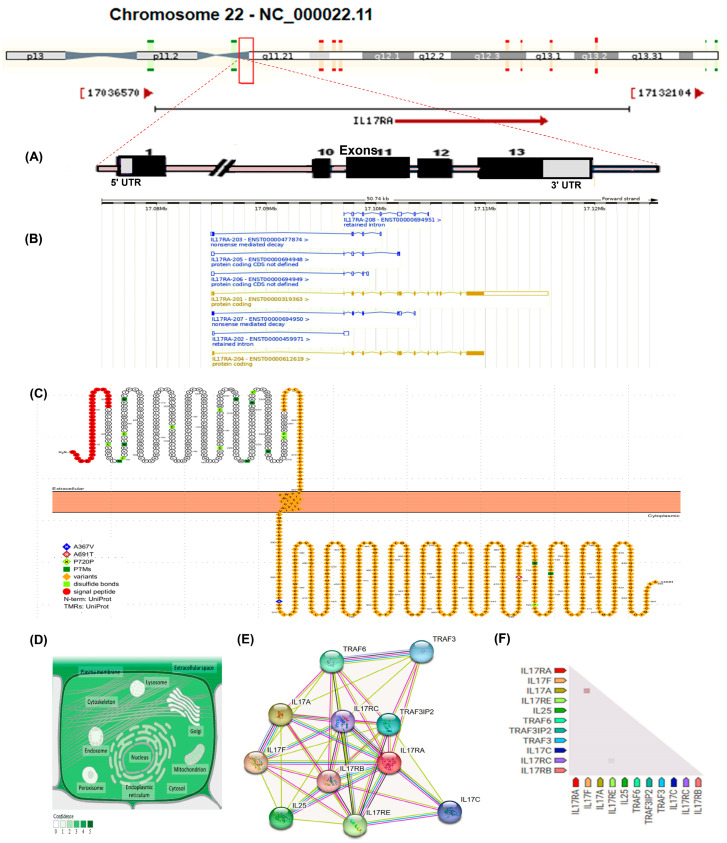
Molecular features of interleukin-17RA (*IL17RA)* gene and protein. (**A**) The *IL17RA* gene (Gene ID: 23765) is located on the long arm of chromosome 22-NC_000022.11:17,084,954-17,115,693 according to Homo sapiens assembly GRCh38.p14, with 30,740 bp long, on the forward strand. It consists of 13 exons. (**B**) The gene has eight transcripts: the major one (IL17RA-201 transcript; ENST00000319363.11) has 8566 nucleotides, which codes for 866aa protein; the IL17RA-204 (2499 nucleotides) codes for the 832aa protein, the IL17RA-207 (739 nucleotides) codes for the129aa nonsense-mediated decay transcript, the IL17RA-203 (651 nucleotides) codes for the 59aa nonsense-mediated decay transcript, and the IL17RA-206, 205, 208, and 202 (675, 611, 847, and 621 nucleotides, respectively) are non-protein coding transcripts. (**C**) The encoded protein is a single polypeptide chain of 866 amino acid residues, starting with the signal peptide (1–32 aa, red color) in the amino terminus, the extracellular domain (33–320aa), the transmembrane domain (321–341aa), and the cytoplasmic domain (342–866aa). The studied missense variants-related amino acid substitutions were labeled in the cytoplasmic domain and explained in the legend with the specified colors. (**D**) The subcellular localization of IL17RA can be observed, with the intensity of green coloration indicating its relative abundance. (**E**) The interacting protein–protein network demonstrates the functional partners of the IL17RA. The generated network consists of 11 nodes (each node corresponds to a protein generated from a unique gene locus) and 45 edges (each edge signifies an association between proteins, which could imply sharing functionality but does not necessarily mean the proteins are physically bound to each other), with average local clustering coefficient = 0.889, and the protein–protein interaction enrichment *p*-value = 4.22 × 10^−15^. (**F**) *IL17RA* gene coexpression triangle matrix. The confidence level of association is demonstrated by the color intensity, given the overall expression data in humans. Data sources: “https://www.genecards.org/, https://www.ncbi.nlm.nih.gov/gene/23765, https://asia.ensembl.org/Homo_sapiens/Tran-script/, https://wlab.ethz.ch/protter/, https://www.uniprot.org/, https://compartments.jensenlab.org/, and https://string-db.org/” (last accessed on 20 July 2023).

The *IL-17 RA* gene (ID: 23765, ENSG00000177663) maps to the long arm of the human chr22 at the locus 22q11.1 spanning 17,084,954 to 17,115,693 on the plus strand (Figure 2A). It has 13 exons and eight transcripts (splice variants), with two codes for proteins (i.e., IL17RA-201/204) (Figure 2B). The cell membrane attached protein consists of 866aa, and the other secretory one consists of 832aa. The cell membrane-attached receptor comprises a signal peptide and the extracellular, transmembrane, and cytoplasmic domains (Figure 2C). The predominant subcellular localization is the cell membrane as a single pass type I protein and the extracellular secretory isoform (Figure 2D). *IL17RA* exhibits substantial co-expression with IL17F, which acts as a ligand for both IL17RA and IL17RC. The heterodimer formed by IL17A/IL17F is a ligand for the heterodimeric receptor complex composed of IL17RA/IL17RC. This interaction is integral in promoting the production of various cytokines, including IL6, IL8, and CSF2 [68]. Furthermore, IL17RA is involved in the activation of peripheral blood mononuclear cells, T-cell proliferation, and the inhibition of angiogenesis. It also plays a crucial role in inducing lung neutrophilia and exacerbating antigen-induced pulmonary allergic inflammation. Additionally, IL17RA is co-expressed with IL17RE, a specific receptor for IL17C, and is implicated in signal transduction via the NF-kappa-B and MAPK pathways, likely requiring TRAF3IP2/ACT1 for this signaling. This receptor may be essential in the innate immune defense against bacterial infections. The second and fourth isoforms of IL17RA may reside in inactive states within the cytoplasm, while the third and fifth isoforms might function as soluble decoy receptors (Figure 2E,F) (https://string-db.org/) (last accessed on 20 July 2023). The *IL17RA* gene ontology (i.e., cellular component, molecular functions, and biological process) is represented in Supplementary Table S2. The impact of the studied *IL17RA* variants on gene expression and/or function is illustrated in Table 10.

## 4. Discussion

Asthma is a chronic condition characterized by immune-mediated inflammation of the airways, leading to airway remodeling, hyperreactivity, increased mucus production, and airflow obstruction. These changes can cause symptoms that range from mild respiratory discomfort to severe, life-threatening respiratory distress [69]. Various cells and cytokines play critical roles in this process. However, the exact contributions of individual cytokines and their genetic variants to the development and severity of pediatric asthma are not yet fully understood [70,71,72].

Extensive research on genetic susceptibility to asthma has primarily targeted genes encoding polymorphic proteins, particularly those regulating immune responses [73]. Over 60 genetic loci have been linked to asthma, and genome-wide association studies (GWAS) in children and adults with asthma have identified several genes associated with immune responses [73,74,75]. These studies also found that some of these genes encode cytokines that act as immune modulators, and variations in these genes may affect one’s susceptibility to asthma [76].

IL17A is one of these cytokines and has been shown to induce structural changes in airway smooth muscle cells, which may affect susceptibility to airway hyperreactivity (AHR) and the development of asthma [77]. Specific genetic variations in IL17A, such as rs2275913 G/A, rs3819024 A/G, and rs8193036 C/T, have been linked to asthma risk. The rs2275913 variant is associated with an increased risk of developing asthma, whereas the rs8193036 variant may have protective effects [77].

The IL17 receptor A-coding gene (*IL17RA*; ENSG00000177663) is highly polymorphic. Several *IL-17RA* variations have been discovered as risky in several disorders’ pathogenesis, most of which are immune-mediated, including autoimmune diabetes [78], psoriasis [79,80,81], chronic spontaneous urticaria (CSU) [82], alopecia areata [83], ankylosing spondylitis [84]. The impact of genetic variation can vary from no effect to alteration of protein expression, processing/maturation, and activity [85].

Evidence suggests a potential link between IL-17RA SNPs and respiratory diseases. Specifically, genetic variants of IL-17R (rs882643 and rs2241049) have been associated with primary graft dysfunction in lung transplantation [86] and aspirin-exacerbated respiratory disease [87,88].

To the authors’ knowledge, this study is the first to interrogate the associations between four *IL17RA* SNPs: rs4819554A/G, rs879577C/T, rs41323645G/A, and rs4819555C/T with asthma susceptibility/phenotype in an Egyptian population.

Currently, the *IL17RA* rs4819554 shows that the G/G genotype reduces disease risk significantly, and the G allele is protective. This association was observed in the dominant/codominant and recessive models compared to the reference A/A genotype. Carriers of the A allele for this variant had a four-fold higher disease risk and demonstrated a significant association with a positive asthma FH and using the reliever for more than two weeks. Analysis of risk allele carriage and association with patient characteristics/disease outcome and therapeutic response refile that risk allele was associated with lower values of two parameters to assess therapeutic response, PEFR1 and post FEV1.

The rs4819554 SNP is a common *IL17RA* variant discovered upstream in the gene (c.-947G > A), and because it is located in the *IL17RA* promoter region, it may have a functional effect by affecting gene transcription. Compared to the other genotypes, the rs4819554 AA genotype enhanced protein production. This genotype may regulate inflammatory responses by boosting the production of TNF and other proinflammatory cytokines [89]. Interestingly, this SNP is in linkage disequilibrium with other promoter polymorphisms that influence transcription factor binding, such as the binding sites for Lyf-1 and Ik-2. These transcription factors are part of the Ikaros (IK) family, which has been associated with Th17 cell differentiation by upregulating the expression of Th17 lineage-determining genes, including IL-17A, IL-17F, IL-21, IL-22, and IL-23R, while also suppressing the expression of genes that inhibit Th17 cell development [90].

The rs4819554 has been related to enhanced vulnerability or protection against various inflammatory and immunological disorders, as well as some types of cancer. P Park et al. observed that this SNP may predispose individuals to asthma by upregulating IL17RA expression. Asthmatics exhibit higher IL-17RA mRNA and protein levels in CD14+ monocytes and mononuclear cells. This upregulation was significantly more pronounced in individuals with the AA genotype than those with the GG genotype, which aligns with the decreased frequency of the minor allele G in asthmatic patients [88].

In a study conducted by Lauhkonen and colleagues to examine the relationship between three IL17RA polymorphisms, including rs4819554, and various outcomes such as asthma, asthma medication use, allergic rhinitis, and lung function decline at 5–7 years and 11–13 years after hospitalization for bronchiolitis in the first six months of infancy, no correlation was found between the IL17RA gene variations studied and susceptibility to severe bronchiolitis in infancy or subsequent post-bronchiolitis asthma [91]. Additionally, autoimmune type 1 diabetes and other inflammatory and autoimmune disorders have been linked to this variation. The GG genotype was discovered to be linked with a lower risk than the AA and AG genotypes and to be negatively correlated with the presence of serum “glutamic acid decarboxylase antibodies” [78].

The rs4819554 SNP was significantly associated with an increased risk of psoriasis. Psoriatic patients exhibited higher frequencies of the G allele and AG genotype than controls. Additionally, patients with AA or AG genotypes had significantly elevated gene transcript and protein levels relative to those in normal controls. This suggests that the AA genotype of rs4819554 may be linked to a reduced likelihood of acquiring psoriasis [79]. The rs48419554 can impact protein biological activity and thus modify immunological aspects such as response to treatment. IL-17RA rs48419554 was previously related to etanercept response in psoriatic arthritis [92]. Using the pharma GKB database to explore clinical annotation, which provides information about variant-drug connects based basically on variant annotations for rs4819554, it was revealed that patients with the AA genotype and psoriasis might have a better response to treatment with anti-TNF drugs than patients with the AG or GG genotype and carriers of the GG genotype have the poorer response (level 3 efficacy) (http://www.pharmgkb.org) (last accessed on 30 July 2023)

Sandip et al. identified a significant association between the rs4819554 variant and cardiovascular mortality in patients with congestive heart failure, with a hazard ratio (HR) of 1.28 (95% CI = 1.02–1.59, adjusted *p* = 0.03) [93]. Another study reported that, in patients with coronary artery disease (CAD), the IL17RA transcript levels were elevated in total leukocytes, and rs4819554*G carriers exhibited higher transcript levels compared to controls despite no significant association being found between the rs4819554 variant and CAD risk [94]. The GG genotype of rs4819554 has also been reported as a risk factor for preeclampsia [95]. Additionally, the G allele and the combined AG + GG genotypes were strongly associated with Hashimoto’s thyroiditis, particularly in cases with a positive family history and increased illness severity [96].

The *IL-17RA* rs48419554 G allele may be a potential marker of disease severity in Polish ankylosing spondylitis patients [97]. Additionally, patients with chronic spontaneous urticaria (CSU) had significantly more GG genotypes than controls, and the frequency of the A allele was notably lower in urticaria patients. The G allele was considered a risk factor. Despite this, patients with the rs4819554 AA genotype were associated with longer disease duration, concurrent angioedema, positive autologous serum skin test status, advanced treatment stages, and the lowest quality of life scores [82]. This may be partially explained by an increase in the level of the IL17RA protein with the A allele, which causes the condition to become more severe [88,98].

Moreover, the rs4819554 variant was significantly associated with worse progression-free survival and overall survival in patients with non-small cell lung cancer (NSCLC) [99]. Similarly, homozygosity for the AA genotype was linked to more significant deterioration of renal function [98] and increased primary graft dysfunction after lung transplantation [86]. Conversely, the rs4819554 AG genotype was associated with smaller tumor size, earlier tumor stage at diagnosis, and a better response to initial therapy [100].

According to 1000 Genomes Project Phase 3, the minor allele (G) frequency of this variant is reported to be “0.008 in Africans, 0.41 in Americans, 0.42 in East Asians, 0.24 in South Asians, and 0.21 in Europe”. A bioinformatic analysis using haploreg4.2 illustrated that this variant was associated with an alternation of six motifs, including AP-4, ASCL2, HEN1, LBP-1, TCF2, VDR theses motifs were related to COPD, inflammation and immunity [101,102,103,104,105,106].

The C to T substitution (c.1100C > T) in exon 13 causes an amino acid shift from alanine to valine at codon 367, resulting in rs879577, another frequent missense variation. This SNP overlaps three IL-17RA transcripts. This variation was associated with CSU; CC genotype and C allele were predominant among patients and associated with poor clinical indicators, concurrent angioedema, leg lesions, and low quality of life [82]. This result may have been caused by substituting the amino acid valine (p.A367V) for alanine (p.A367A) in the IL-17RA cytoplasmic domain. Because the two amino acids have different structural and biochemical prosperities, this substitution may have altered the protein’s conformation and had functional effects.

In the Chinese population, Li et al. found an insignificant relationship between this variation and autoimmune diabetes type 1 [78]. According to Batalla et al., no conclusive evidence exists that rs879577 increases the likelihood of developing psoriasis [80]. The rs879577 variant was highly enriched in Korean alopecia areata patients [107]. The bioinformatics online tool “I-Mutant v.2 (available at https://folding.biofold.org/i-mutant//pages/I-Mutant2.0_Details.html),” accessed on 20 July 2023, shows that this variation results in higher protein stability, and Polyphen-2 v2.2.2, which predicts the functional effect of the variant on proteins, shows that this variant is benign and tolerated.

The minor allele (A) frequency among different ethnicities was found to be “0.47 in Africans, 0.28 in Americans, 0.09 in East Asians, 0.19 in South Asians, and 0.26 in Europe (www.ensembl.org)”, last accessed on 20 July 2023. QLT bioinformatic analysis revealed this polymorphism showed linkage disequilibrium with other 4 SNP on *IL17RA* rs34484815, rs879575, rs4819555, rs1468488 and was shown to be associated with four altered motifs including AP-2, ER alpha-a, Rad 21, and sin3AK-20. Some have been reported previously to be associated with oxidant responses, COPD, and asthma [108,109,110].

In our study population, this variant was found to reduce disease risk, and the minor allele (T) was shown to decrease risk across all models, similar to rs4819554, the reference allele of rs879577 C was associated with higher disease risk, and T allele was associated with a protective effect, further analysis regarding carrying risk allele and disease severity, outcome, allergies, and aggravating factors, therapeutic response reveal non-significant association.

The third investigated variant in this study was *IL17RA*_rs41323645G/A. This missense variant overlaps three transcripts, resulting in a different amino acid sequence but where the length is preserved A (Ala) > T (Thr). Ala691Thr. I-Mutant v.2 online tool, this variation results in decreased protein stability, and Polyphen-2 v2.2.2 shows that this variant is predicted to be probably damaging. The minor allele (A) frequency is reported to be 0.24 in Africans, 0.8 in Americans, not present in East Asians, 0.10 in South Asians, and 0.11 in Europe. QLT analysis reveals rs41323645 is in linkage disequilibrium with rs73381911, rs879576, rs57380532, rs11702918, and rs11703539 on *IL-17RA* and was associated with changes in neuron-restrictive silencer factor (NRSF) motif which shown to exert inhibitory effects on cell proliferation/migration of lung squamous cell carcinoma [111]. To our knowledge, this variation has never been studied in asthma. This mutation, along with variants in the *NOD2*, *EPHA2*, and *KALRN* genes, may play an important role in the emergence of sarcoidosis by sustaining a persistent proinflammatory condition in macrophages [112]. Our findings show that the A allele was more common in patients than controls and was associated with increased disease risk in all models. In contrast, the G allele appears to be protective, and further analysis of the association between the A allele and disease severity, outcome, therapeutic response, and allergy reveals non-significance except for the use of asthma reliever for more than two weeks and risk factors as emotional stress triggers and smoking exposure.

*IL17RA*_rs4819555C/T (c.2160C > T) (p.Pro720) synonymous variant has a frequency of the minor allele (T) reported to be “0.38 in Africans, 0.27 in Americans, 0.10 in East Asians, 0.18 in South Asians, and 0.25 in Europe (www.ensemble.org),” last accessed on 20 July 2023. In the current study, an eQTL bioinformatic analysis showed that rs4819555 was found to be in linkage disequilibrium with nearby SNPs, such as rs879575 and rs879577, which could also be potential candidates associated with asthma risk. Furthermore, the rs4819555 variant could influence binding with the transcription factors BDP1, EBF, HNF4, NRSF, PLAG1, STAT, and ZNF263, which are linked to carcinogenesis, inflammation, and immunity [113,114,115,116,117]. Although a trend of increased disease risk with the minor (T) allele was observed, there was an insignificant association with disease risk in our study. However, carriers of the T allele significantly correlate with atopic asthma and comorbidities such as allergic rhinitis and eczema. There was also a significant difference in eosinophilic count between carriers and non-carriers.

It is worth noting that the discrepancy between SNPs, either risk or protection in the literature and the current study is caused by the recognized genetic heterogeneity, different populations, ethnicity, various environmental factors, geographic distribution, variations in sample size/study methodology, and the underlying effects that genetic variants cause in a cell/context-specific manner.

Increasing understanding of genetic disease susceptibility, it has been found that considering combined genotypes, or haplotypes, in association studies is far more insightful and compelling for identifying risk related to asthma than relying solely on a single polymorphism [118,119]. The reason for this is the complexity of human genetic composition, in which many diseases, including asthma, present as multifactorial disorders involving more than a single gene variant [120,121]. Consequentially, a risk variant in isolation can provide limited information and be misleading, providing an inadequate understanding of disease susceptibility. Accordingly, in the current study, we found that the most frequent haplotype in the asthma group was ACAC, with a frequency of 22.87% in the patient group vs. 1.36% in controls, and this haplotype was associated with increased asthma risk (Table 5). Also, other haplotypes, including ACGC, ATGC, and GCAC, were significantly associated with asthma, albeit with lower frequencies than ACAC. Although these identified haplotypes were not associated with asthma severity or treatment response, they allow for a more comprehensive and accurate understanding of asthma risk. It embodies the concept of cumulative effect from multiple gene variations. Therefore, the panoramic perspective obtained by studying the interactions of multiple genetic variants confers a more sophisticated understanding of the relationships between gene combinations and disease risk, ultimately leading to enhanced predictive ability and improved personalized medicine strategies.

Although this is the first to highlight the association between *IL17RA* SNPs rs4819554, rs879577, rs41323645, and rs4819555 and asthma, various limitations should be addressed. The sample was relatively small, and there was an unavoidable selection bias as both patients and controls were enrolled from the same hospital. Functional analyses for the studied variants are required to elucidate the precise mechanism by which these variants confer asthma susceptibility/protection; thus, further research is needed to investigate variant effects in large populations with diverse ethnic backgrounds supported by including more *IL17RA*-related SNPs through rigorous genetic matching and functional analysis. Furthermore, future studies investigating the potential influence of the identified SNPs on serum biomarkers are highly recommended.

## 5. Conclusions

In conclusion, the present results indicate a significant association of *IL17RA* polymorphisms rs4819554, rs879577, and rs41323645 with disease risk under different genetic models and the predominance of the risk haplotype ACAC in the patient group. Meanwhile, the rs4819555 variant showed associations with different phenotypic features and comorbidities of the studied population. These findings need to be validated in larger and more diverse populations.

## Figures and Tables

**Table 1:** TaqMan assay-related data applied in the present study.

SNP Reference Sequence	Assay ID	Location on Ch.22 According to GRCh38 Assembly	Context Sequence [VIC/FAM]
rs4819554	C__337392_30	17084145	GGGAAGTAACGACTCTCTTAGGTGC[A/G]GC TGGGACACAGTCTCACAGACCAG
rs879577	C__2666446_20	17108319	CTTGTTTCCTTAGATGGCCTGCCTG[C/T]GGC TGACCTGATCCCCCCACCGCTG
rs41323645	C__86401686_10	17109290	GCCTGGGCCCCTGGCTGACGGTGCC[A/G]CA GTCCGGCTGGCACTGGCGGGGG
rs4819555	C__28000962_10	17109379	GGCGAAATAGCGTCCTCTTCCTCCC[C/T]GTG GACCCCGAGGACTCGCCCCTTG

All variants are of the “transition substitution” type. All assays have the catalog number 4351379 (Applied Biosystems, ThermoFisher Scientific, Waltham, MA, USA) (https://www.thermofisher.com/), last accessed on 20 July 2023. The ancestral/minor alleles of each studied variant labeled by VIC/FAM probes are blue-colored in the context gene sequence.

**Table 2:** Baseline characteristics of the study population.

Characteristics	Levels	Control (*n* = 96)	Asthma (*n* = 96)	*p*-Value
Age	Mean ± SD	9.6 ± 3.1	9.7 ± 3.0	0.76
Sex	Female	56 (58.3%)	52 (54.2%)	0.66
	Male	40 (41.7%)	44 (45.8%)	
Residency	Rural	51 (53.1%)	64 (66.7%)	0.08
	Urban	45 (46.9%)	32 (33.3%)	
FH of asthma	Negative	89 (92.7%)	67 (69.8%)	**<0.001**
	Positive	7 (7.3%)	29 (30.2%)	
BMI %	<85th	51 (53.1%)	53 (55.2%)	0.95
	<95th	34 (35.4%)	32 (33.3%)	
	≥95th	11 (11.5%)	11 (11.5%)	
Pubertal status	Negative	52 (54.2%)	51 (53.1%)	0.88
	Positive	44 (45.8%)	45 (46.9%)	
Tanner stage *	Stage 1	52 (54.2%)	51 (53.1%)	0.65
	Stage 2	14 (14.6%)	17 (17.7%)	
	Stage 3	18 (18.8%)	12 (12.5%)	
	Stage 4	6 (6.3%)	10 (10.4%)	
	Stage 5	6 (6.3%)	6 (6.3%)	

Data (except age) are reported as frequency/percentage. A Chi-Square test (two-sided) was employed. The bold value indicates *p*-value < 0.05. * According to “Marshall and Tanner” [53]. FH: family history, BMI%: body mass index percentile.

**Table 3:** Genotype frequency and genetic association model.

Genetic Models	Genotype	Controls (*n*= 96)	Patients (*n* = 96)	OR (95%CI)	*p*-Value	AIC
rs4819554						
Codominant Model	A/A	29 (30.2%)	47 (49%)	1	**0.001**	250.4
	A/G	44 (45.8%)	42 (43.8%)	0.69 (0.35–1.36)		
	G/G	23 (24%)	7 (7.3%)	**0.15 (0.05–0.45)**		
Dominant Model	A/A	29 (30.2%)	47 (49%)	1	**0.028**	257.4
	A/G-G/G	67 (69.8%)	49 (51%)	**0.49 (0.26–0.93)**		
Recessive Model	A/A-A/G	73 (76%)	89 (92.7%)	1	**<0.001**	**249.6**
	G/G	23 (24%)	7 (7.3%)	**0.18 (0.07–0.52)**		
Over dominant	A/A-G/G	52 (54.2%)	54 (56.2%)	1	0.73	262
	A/G	44 (45.8%)	42 (43.8%)	1.12 (0.60–2.07)		
rs879577						
Codominant Model	C/C	23 (24%)	57 (59.4%)	1	**<0.001**	237.3
	C/T	48 (50%)	31 (32.3%)	**0.22 (0.10–0.45)**		
	T/T	25 (26%)	8 (8.3%)	**0.13 (0.05–0.36)**		
Dominant Model	C/C	23 (24%)	57 (59.4%)	1	**<0.001**	**236.2**
	C/T-T/T	73 (76%)	39 (40.6%)	**0.19 (0.10–0.37)**		
Recessive Model	C/C-C/T	71 (74%)	88 (91.7%)	1	**0.004**	253.8
	T/T	25 (26%)	8 (8.3%)	**0.28 (0.11–0.70)**		
Over dominant	C/C-T/T	48 (50%)	65 (67.7%)	1	**0.003**	253.1
	C/T	48 (50%)	31 (32.3%)	**0.37 (0.19–0.72)**		
rs41323645						
Codominant Model	G/G	42 (43.8%)	23 (24%)	1	**0.004**	253
	G/A	39 (40.6%)	43 (44.8%)	**2.47 (1.19–5.14)**		
	A/A	15 (15.6%)	30 (31.2%)	**3.86 (1.62–9.18)**		
Dominant Model	G/G	42 (43.8%)	23 (24%)	1	**0.002**	**252.1**
	G/A-A/A	54 (56.2%)	73 (76%)	**2.89 (1.47–5.68)**		
Recessive Model	G/G-G/A	81 (84.4%)	66 (68.8%)	1	**0.025**	257.1
	A/A	15 (15.6%)	30 (31.2%)	**2.34 (1.10–4.98)**		
Over dominant	G/G-A/A	57 (59.4%)	53 (55.2%)	1	0.24	260.8
	G/A	39 (40.6%)	43 (44.8%)	1.45 (0.78–2.72)		
rs4819555						
Codominant Model	C/C	42 (43.8%)	33 (34.4%)	1	0.19	260.9
	C/T	42 (43.8%)	45 (46.9%)	1.43 (0.72–2.82)		
	T/T	12 (12.5%)	18 (18.8%)	2.32 (0.91–5.92)		
Dominant Model	C/C	42 (43.8%)	33 (34.4%)	1	0.14	260
	C/T-T/T	54 (56.2%)	63 (65.6%)	1.61 (0.85–3.07)		
Recessive Model	C/C-C/T	84 (87.5%)	78 (81.2%)	1	0.13	**259.9**
	T/T	12 (12.5%)	18 (18.8%)	1.91 (0.81–4.47)		
Over dominant	C/C-T/T	54 (56.2%)	51 (53.1%)	1	0.75	262.1
	C/T	42 (43.8%)	45 (46.9%)	1.11 (0.60–2.05)		

Data are reported as frequency/percentage. A Chi-Square test (two-sided) was employed. Logistic binary regression was carried out, adjusted by age, sex, residency (rural vs. urban), family history, BMI percentile, and puberty defined according to “Marshall and Tanner” [53]. The table also provides the odds ratio (OR) with its 95% confidence interval (CI), the *p*-value, and the Akaike information criterion (AIC) for each model. Bold values indicate *p*-value < 0.05.

**Table 4:** The carriage rate of alleles and disease risk.

Allele	Overall	Controls	Patients	OR (95%CI)	*p*-Value
rs4819554					
A	162 (84.4%)	73 (76%)	89 (92.7%)	4.01 (1.63–9.86)	**0.002**
G	116 (60.4%)	67 (69.8%)	49 (51%)	0.45 (0.25–0.82)	**0.012**
rs879577					
C	159 (82.8%)	71 (74%)	88 (91.7%)	3.87 (1.65–9.11)	**0.002**
T	112 (58.3%)	73 (76%)	39 (40.6%)	0.22 (0.12–0.4)	**<0.001**
rs41323645					
G	147 (76.6%)	81 (84.4%)	66 (68.8%)	0.41 (0.2–0.82)	**0.016**
A	127 (66.1%)	54 (56.3%)	73 (76%)	2.47 (1.33–4.58)	**0.006**
rs4819555					
C	162 (84.4%)	84 (87.5%)	78 (81.3%)	0.62 (0.28–1.37)	0.32
T	117 (60.9%)	54 (56.3%)	63 (65.6%)	1.48 (0.83–2.66)	0.24

Data are presented as frequency and percentage. A Chi-Square test (two-sided) was employed. Logistic binary regression was carried out. Unadjusted odds ratio (OR) with its 95% confidence interval (CI) is calculated for each allele in the patient group, using the control group as a reference. Bold values indicate *p*-values < 0.05.

**Table 5:** Subgroup analysis by puberty. Haplotype frequency analysis.

SNP ID	Genotype	Prepuberty			Puberty			*p*-Value *
		Controls	Patients	OR (95%CI)	Controls	Patients	OR (95%CI)	
rs4819554	A/A	29	47	1	14	19	1	0.33
	A/G-G/G	67	49	**0.49 (0.26–0.93)**	30	26	0.70 (0.27–1.77)	
rs879577	C/C	11	32	1	12	25	1	0.13
	C/T-T/T	41	19	**0.11 (0.04–0.30)**	32	20	**0.33 (0.13–0.87)**	
rs41323645	G/G	22	10	1	20	13	1	0.65
	G/A-A/A	30	41	**3.35 (1.30–8.62)**	24	32	2.47 (0.95–6.43)	
rs4819555	C/C	26	19	1	16	14	1	0.91
	C/T-T/T	26	32	1.67 (0.72–3.84)	28	31	1.55 (0.58–4.11)	

* The *p*-values presented in the last column test for interaction in the trend, which indicates if the effect of the genotype on disease risk changes with puberty status. Bold values indicate a statistical significance at *p*-value < 0.05.

**Table 6:** Subgroup analysis by puberty. Haplotype frequency analysis.

	Haplotype	Total	Controls	Patients	Cumulative Frequency	OR (95%CI)	*p*-Value
1	ACAC	0.1391	0.0136	0.2287	0.1391	1	---
2	ACGC	0.1332	0.1517	0.1472	0.2723	**0.00 (0.00–0.11)**	**0.002**
3	ATGC	0.1016	0.1777	0.0218	0.3739	**0.00 (0.00–0.05)**	**<0.001**
4	GCAC	0.0882	0.0745	0.0683	0.4621	**0.00 (0.00–0.08)**	**0.002**
5	ACGT	0.0879	0.1064	0.0923	0.55	**0.02 (0.00–0.36)**	**0.009**
6	ATGT	0.0701	0.0784	0.0725	0.6201	**0.00 (0.00–0.07)**	**0.001**
7	GCGC	0.0503	0.0499	0.0502	0.6704	0.06 (0.00–1.46)	0.08
8	GTGC	0.0499	0.0528	0.0294	0.7203	0.00 (-Inf–Inf)	1.00
9	ACAT	0.0482	0.092	0.0162	0.7685	**0.02 (0.00–0.48)**	**0.018**
10	GTAC	0.0446	0.0584	0.0034	0.8131	**0.00 (0.00–0.04)**	**<0.001**
11	ATAT	0.0426	0.0288	0.0725	0.8556	NA	**<0.001**
12	GCAT	0.0353	0.0593	0	0.8909	**0.00 (0.00–0.14)**	**0.003**
13	GCGT	0.0329	0.0381	0.0336	0.9237	0.03 (0.00–1.01)	0.05
14	GTAT	0.0327	NA	0.079	0.9564	**0.00 (0.00–0.12)**	**0.004**
15	GTGT	0.0313	0.012	0.0665	0.9877	**0.00 (0.00–0.16)**	**0.004**
16	ATAC	0.0123	0.0066	0.0182	1	0.00 (0.00–1.36)	0.06

Bold values indicate a statistical significance of the observed differences at *p*-value < 0.05.

**Table 7:** Association of carriage of risky allele on patient characteristics and disease outcomes.

Characteristics		rs4819554*A Carrier		*p*-Value	rs879577*C Carrier		*p*-Value	rs41323645*A Carrier		*p*-Value	rs4819555*C Carrier		*p*-Value
		No	Yes		No	Yes		No	Yes		No	Yes	
Total number		7	89		8	88		23	73		18	78	
Demographics													
Age	Mean ± SD	9.6 ± 2.9	9.7 ± 3.1	0.84	9.5 ± 3.3	9.7 ± 3	0.70	9.9 ± 3.1	9.6 ± 3.1	0.48	10.4 ± 3.3	9.6 ± 3	0.16
Sex	Male	3 (42.9%)	41 (46.1%)	0.87	4 (50%)	40 (45.5%)	0.80	12 (52.2%)	32 (43.8%)	0.63	6 (33.3%)	38 (48.7%)	0.30
Residency	Urban	4 (57.1%)	28 (31.5%)	0.22	5 (62.5%)	27 (30.7%)	0.11	5 (21.7%)	27 (37%)	0.21	8 (44.4%)	24 (30.8%)	0.28
FH of asthma	Positive	5 (71.4%)	24 (27%)	**0.025**	2 (25%)	27 (30.7%)	0.74	7 (30.4%)	22 (30.1%)	0.98	4 (22.2%)	25 (32.1%)	0.57
Pubertal status	Positive	5 (71.4%)	40 (44.9%)	0.25	4 (50%)	41 (46.6%)	0.85	13 (56.5%)	32 (43.8%)	0.34	7 (38.9%)	38 (48.7%)	0.60
BMI %	<85th	2 (28.6%)	51 (57.3%)	0.21	3 (37.5%)	50 (56.8%)	0.38	12 (52.2%)	41 (56.2%)	0.76	10 (55.6%)	43 (55.1%)	1.00
	<95th	3 (42.9%)	29 (32.6%)		3 (37.5%)	29 (33%)		9 (39.1%)	23 (31.5%)		6 (33.3%)	26 (33.3%)	
	≥95th	2 (28.6%)	9 (10.1%)		2 (25%)	9 (10.2%)		2 (8.7%)	9 (12.3%)		2 (11.1%)	9 (11.5%)	
Presentation													
Onset	Early (≤3 y)	2 (28.6%)	43 (48.3%)	0.44	5 (62.5%)	40 (45.5%)	0.47	10 (43.5%)	35 (47.9%)	0.81	10 (55.6%)	35 (44.9%)	0.44
	Late (>3 y)	5 (71.4%)	46 (51.7%)		3 (37.5%)	48 (54.5%)		13 (56.5%)	38 (52.1%)		8 (44.4%)	43 (55.1%)	
Asthma phenotype	Atopic	6 (85.7%)	70 (78.7%)	0.67	4 (50%)	72 (81.8%)	0.10	19 (82.6%)	57 (78.1%)	0.53	11 (61.1%)	65 (83.3%)	**0.007**
	Non-atopic	1 (14.3%)	6 (6.7%)		2 (25%)	5 (5.7%)		2 (8.7%)	5 (6.8%)		3 (16.7%)	4 (5.1%)	
	Exercise-induced	0 (0%)	11 (12.4%)		2 (25%)	9 (10.2%)		1 (4.3%)	10 (13.7%)		2 (11.1%)	9 (11.5%)	
	Aspirin-sensitive	0 (0%)	2 (2.2%)		0 (0%)	2 (2.3%)		1 (4.3%)	1 (1.4%)		2 (11.1%)	0 (0%)	
Symptoms	Cough	7 (100%)	87 (97.8%)	0.69	8 (100%)	86 (97.7%)	0.67	22 (95.7%)	72 (98.6%)	0.42	18 (100%)	76 (97.4%)	0.49
	Dyspnea	3 (42.9%)	53 (59.6%)	0.45	4 (50%)	52 (59.1%)	0.72	17 (73.9%)	39 (53.4%)	0.09	12 (66.7%)	44 (56.4%)	0.60
	Sputum	3 (42.9%)	51 (57.3%)	0.70	3 (37.5%)	51 (58%)	0.29	16 (69.6%)	38 (52.1%)	0.16	9 (50%)	45 (57.7%)	0.60
	Tightness	4 (57.1%)	60 (67.4%)	0.68	6 (75%)	58 (65.9%)	0.71	16 (69.6%)	48 (65.8%)	0.80	16 (88.9%)	48 (61.5%)	**0.029**
	Wheezes	4 (57.1%)	76 (85.4%)	0.09	6 (75%)	74 (84.1%)	0.62	20 (87%)	60 (82.2%)	0.75	14 (77.8%)	66 (84.6%)	0.49
	Daytime symptoms > 2 wk.	2 (28.6%)	46 (51.7%)	0.44	5 (62.5%)	43 (48.9%)	0.71	13 (56.5%)	35 (47.9%)	0.63	12 (66.7%)	36 (46.2%)	0.19
	Night awakening	0 (0%)	16 (18%)	0.60	0 (0%)	16 (18.2%)	0.34	4 (17.4%)	12 (16.4%)	0.91	2 (11.1%)	14 (17.9%)	0.73
	Reliever use > 2 wks.	1 (14.3%)	32 (36%)	0.42	1 (12.5%)	32 (36.4%)	0.26	12 (52.2%)	21 (28.8%)	**0.047**	6 (33.3%)	27 (34.6%)	0.92
	Activity limitations	2 (28.6%)	27 (30.3%)	0.92	3 (37.5%)	26 (29.5%)	0.69	10 (43.5%)	19 (26%)	0.13	7 (38.9%)	22 (28.2%)	0.40
Comorbidities	Positive	2 (28.6%)	46 (51.7%)	0.44	3 (37.5%)	45 (51.1%)	0.72	15 (65.2%)	33 (45.2%)	0.15	3 (16.7%)	45 (57.7%)	**0.003**
	Allergic rhinitis	2 (28.6%)	36 (40.4%)	0.70	2 (25%)	36 (40.9%)	0.47	11 (47.8%)	27 (37%)	0.46	3 (16.7%)	35 (44.9%)	**0.033**
	Eczema	0 (0%)	30 (33.7%)	0.09	2 (25%)	28 (31.8%)	0.69	9 (39.1%)	21 (28.8%)	0.44	0 (0%)	30 (38.5%)	**0.001**
Asthma Severity	Mild	5 (71.4%)	35 (39.3%)	0.20	5 (62.5%)	35 (39.8%)	0.45	10 (43.5%)	30 (41.1%)	0.66	7 (38.9%)	33 (42.3%)	0.96
	Moderate	2 (28.6%)	38 (42.7%)		2 (25%)	38 (43.2%)		8 (34.8%)	32 (43.8%)		8 (44.4%)	32 (41%)	
	Severe	0 (0%)	16 (18%)		1 (12.5%)	15 (17%)		5 (21.7%)	11 (15.1%)		3 (16.7%)	13 (16.7%)	
Duration, years	Median (IQR)	6 (4–9)	5 (5–8)	0.88	5.5 (5–7.8)	5 (5–8)	0.84	6 (5–9)	5 (4–8)	0.16	6 (5–9)	5 (4–8)	0.08
Asthma Control	Well-controlled	5 (71.4%)	30 (34.9%)	0.13	3 (37.5%)	32 (37.6%)	0.38	5 (22.7%)	30 (42.3%)	0.13	4 (23.5%)	31 (40.8%)	0.24
	Partly-controlled	2 (28.6%)	41 (47.7%)		5 (62.5%)	38 (44.7%)		11 (50%)	32 (45.1%)		11 (64.7%)	32 (42.1%)	
	Uncontrolled	0 (0%)	15 (17.4%)		0 (0%)	15 (17.6%)		6 (27.3%)	9 (12.7%)		2 (11.8%)	13 (17.1%)	
Investigations													
Lab tests	High total IgE	3 (42.9%)	33 (37.1%)	0.76	2 (25%)	34 (38.6%)	0.71	11 (47.8%)	25 (34.2%)	0.32	9 (50%)	27 (34.6%)	0.28
	Eosinophilia	3 (42.9%)	11 (12.4%)	0.06	1 (12.5%)	13 (14.8%)	0.86	4 (17.4%)	10 (13.7%)	0.74	5 (27.8%)	9 (11.5%)	0.08
	Total IgE (IU/mL)	75 (25–245)	50 (15–120)	0.33	43.5 (17.5–105)	60 (15–120)	0.69	80 (15–180)	45 (15–120)	0.11	100 (25–155.8)	47.5 (15–120)	0.07
	Eosinophil (×10^6^/L)	240 (20–689)	78 (25–235)	0.43	111 (21.3–243)	78 (25–240)	0.96	78 (25–340)	98 (25–240)	0.85	230 (86–509.8)	50 (20–207.5)	**0.009**

Data (except age) are reported as frequency or median (interquartile range). Chi-Square (two-sided), T-test, or Mann–Whitney U-tests were applied. SD: standard deviation, FH: family history, BMI%: body mass index percentile. Bold values indicate a statistical significance of the observed differences at *p*-value < 0.05.

**Table 8:** Association of carriage of risky allele on patient characteristics and disease outcomes.

Characteristics		rs4819554*A Carrier		*p*-Value	rs879577*C Carrier		*p*-Value	rs41323645*A Carrier		*p*-Value	rs4819555*C Carrier		*p*-Value
		No	Yes		No	Yes		No	Yes		No	Yes	
Total number		30	162		33	159		65	127		30	162	
Management													
AHR	Normal	3 (42.9)	37 (41.6)	0.77	4 (50)	36 (40.9)	0.74	7 (30.4)	33 (45.2)	0.66	4 (22.2)	36 (46.2)	0.14
	Borderline	3 (42.9)	25 (28.1)		3 (37.5)	25 (28.4)		8 (34.8)	20 (27.4)		9 (50)	19 (24.4)	
	Mild/moderate	1 (14.3)	23 (25.8)		1 (12.5)	23 (26.1)		7 (30.4)	17 (23.3)		4 (22.2)	20 (25.6)	
	Severe	0 (0)	4 (4.5)		0 (0)	4 (4.5)		1 (4.3)	3 (4.1)		1 (5.6)	3 (3.8)	
Therapy Level	Step 1	3 (42.9)	18 (20.2)	0.23	2 (25)	19 (21.6)	0.59	4 (17.4)	17 (23.3)	0.91	3 (16.7)	18 (23.1)	0.92
	Step 2	2 (28.6)	17 (19.1)		3 (37.5)	16 (18.2)		6 (26.1)	13 (17.8)		4 (22.2)	15 (19.2)	
	Step 3	2 (28.6)	14 (15.7)		1 (12.5)	15 (17)		4 (17.4)	12 (16.4)		4 (22.2)	12 (15.4)	
	Step 4	0 (0)	32 (36)		1 (12.5)	31 (35.2)		7 (30.4)	25 (34.2)		6 (33.3)	26 (33.3)	
	Step 5	0 (0)	8 (9)		1 (12.5)	7 (8)		2 (8.7)	6 (8.2)		1 (5.6)	7 (9)	
Therapeutic responsePFT	FVC1 (% predicted)	83 (78–89)	80 (72–82.5)	0.07	80 (70.5–88.5)	80 (72–83)	0.66	80 (72–84)	80 (72–82.5)	0.82	81 (77.5–84)	78 (72–82)	0.16
	Pre FEV1 (% predicted)	66 (62–80)	62 (53–72)	0.11	70 (48–85.5)	62 (55–71.5)	0.56	62 (50–76)	62 (59–74)	0.41	61.5 (52–72)	62 (58–76)	0.73
	Post FEV1 (% predicted)	82 (80–88)	80 (70–82)	**0.045**	81.5 (71–89.5)	80 (70.5–83.5)	0.45	78 (70–84)	80 (71–83)	0.96	81 (74–86)	80 (70–82)	0.17
	PEFR1 (%predicted)	86 (80–93)	78 (68–86)	**0.046**	85 (66.5–89)	79 (68–86)	0.39	80 (68–86)	80 (68–86)	0.90	85 (71–88)	79 (68–84.5)	0.22
	PC20	8 (5–8)	4 (1–8)	0.55	8 (2.8–8)	4 (1.8–8)	0.82	6 (1–8)	6 (4–8)	0.69	8 (1–16)	4 (3.3–8)	0.36
	BDRBASE (ml)	32.3 (5.1–33.3)	32.3 (5.4–39.7)	0.34	19 (4.7–47.6)	32.3 (5.6–38.6)	0.87	34.5 (7–42.3)	31.3 (5.2–37.9)	0.24	32.6 (22.1–41.1)	29.2 (5.2–38.5)	0.26

Data are reported as frequency or median (interquartile range). Chi-Square (two-sided) or Mann–Whitney U-tests were applied. “AHR: airway hyperresponsiveness, PFT: pulmonary function test, FVC1: forced vital capacity in 1 s, FEV1: forced expiratory volume in 1 s, PEFR1: peak expiratory flow rate, PC20: provocative concentration causing a 20% drop in FEV1, BDRBASE: bronchodilator response”. Bold values indicate significance at *p*-value < 0.05.

**Table 9:** Impact of the risky haplotype block (rs4819554*A/ rs879577*C/ rs41323645*A) on disease outcomes.

Characteristics		ACA(C/T) Haplotype		*p*-Value
		No	Yes	
Total number		36	60	
Demographics				
Age	Mean ± SD	9.7 ± 3.1	9.6 ± 3.0	0.79
Sex	Male	17 (47.2%)	27 (45%)	0.84
Residency	Urban	12 (33.3%)	20 (33.3%)	1.00
FH of asthma	Positive	12 (33.3%)	17 (28.3%)	0.65
Pubertal status *	Positive	20 (55.6%)	25 (41.7%)	0.21
BMI %	<85th	17 (47.2%)	36 (60%)	0.34
	<95th	13 (36.1%)	19 (31.7%)	
	≥95th	6 (16.7%)	5 (8.3%)	
Presentation				
Onset	Early (≤3 y)	17 (47.2%)	28 (46.7%)	0.96
	Late (>3 y)	19 (52.8%)	32 (53.3%)	
Asthma phenotype	Atopic	27 (75%)	49 (81.7%)	0.24
	Non-atopic	5 (13.9%)	2 (3.3%)	
	Exercise-induced	3 (8.3%)	8 (13.3%)	
	Aspirin-sensitive	1 (2.8%)	1 (1.7%)	
Symptoms	Cough	35 (97.2%)	59 (98.3%)	0.71
	Dyspnea	22 (61.1%)	34 (56.7%)	0.83
	Sputum	20 (55.6%)	34 (56.7%)	0.92
	Tightness	24 (66.7%)	40 (66.7%)	1.00
	Wheezes	28 (77.8%)	52 (86.7%)	0.27
	Daytime Symptoms > 2 wks.	18 (50%)	30 (50%)	1.00
	Night awakening	4 (11.1%)	12 (20%)	0.40
	Reliever use > 2 wks.	12 (33.3%)	21 (35%)	0.87
	Activity limitations	13 (36.1%)	16 (26.7%)	0.36
Comorbidities	Positive	20 (55.6%)	28 (46.7%)	0.53
	Allergic rhinitis	15 (41.7%)	23 (38.3%)	0.83
	Eczema	11 (30.6%)	19 (31.7%)	0.91
Asthma severity	Mild	18 (50%)	22 (36.7%)	0.38
	Moderate	12 (33.3%)	28 (46.7%)	
	Severe	6 (16.7%)	10 (16.7%)	
Asthma control	Well-controlled	13 (37.1%)	22 (37.9%)	0.98
	Partly-controlled	16 (45.7%)	27 (46.6%)	
	Uncontrolled	6 (17.1%)	9 (15.5%)	
Management				
AHR	Normal	12 (33.3%)	28 (46.7%)	0.38
	Borderline	14 (38.9%)	14 (23.3%)	
	Mild/moderate	9 (25%)	15 (25%)	
	Severe	1 (2.8%)	3 (5%)	
Therapy level	Step 1	7 (19.4%)	14 (23.3%)	0.21
	Step 2	11 (30.6%)	8 (13.3%)	
	Step 3	7 (19.4%)	9 (15%)	
	Step 4	8 (22.2%)	24 (40%)	
	Step 5	3 (8.3%)	5 (8.3%)	
Lab tests	High IgE	16 (44.4%)	20 (33.3%)	0.29
	Eosinophilia	8 (22.2%)	6 (10%)	0.14
Therapeutic response				
PFT	FVC1 (% predicted)	80 (72–84)	79 (72–82)	0.32
	Pre FEV1 (% predicted)	62 (52–77.5)	62 (58–71.5)	1.0
	Post FEV1 (% predicted)	80.5 (72.5–86)	79 (70–82)	0.23
	PEFR1 (% predicted)	83 (68.5–87.5)	78 (68–84)	0.16
	PC20	8.0 (1.0–8.0)	4.0 (4.0–8.0)	0.91
	BDRBASE (mL)	32.3 (6.0–41.6)	29.1 (5.2–38.3)	0.39

Data (except age) are reported as frequency or median (interquartile range). Chi-Square (two-sided), T-test, or Mann–Whitney U-tests were applied. “SD: standard deviation, FH: family history, BMI: body mass index, AHR: airway hyperresponsiveness, IgE: immunoglobulin E, PFT: pulmonary function test, FVC1: forced vital capacity in 1 s, FEV1: forced expiratory volume in 1 s, PEFR1: peak expiratory flow rate, PC20: provocative concentration causing a 20% drop in FEV1, BDRBASE: bronchodilator response, SD: standard deviation”. * According to “Marshall and Tanner” [53].

**Table 10:** In silico analysis of the impact of the studied *IL17RA* variants.

SNP ID and Position	Amino Acid change	Region	Mutation Taster	SIFT Prediction (Score)	PANTHER	3D Protein Viewer with a Specified Amino Acid Substitution
rs4819554 c.-947A > G	NA	Promoter 808 bp 5′ of the gene	NA	NA	NA	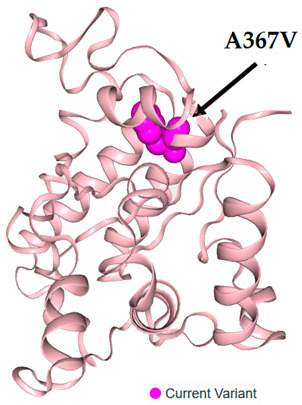
rs879577 c.1100C > A g.23366C > A	A367V	CDS (Exon 13)	Likely benign	Tolerated 0.285	Probably benign	
rs41323645 c.2071G > A g.24337G > A	A691T	CDS (Exon 13)	Benign	Tolerated 0.362	Probably benign	
rs4819555 c.2160C > G	P720P	CDS (Exon 13)	Likely benign	Tolerated 1.0	Possibly damaging	

SNP: single nucleotide polymorphism, SIFT: sorting intolerant from tolerant, PANTHER: Protein ANalysis THrough Evolutionary Relationships, A (amino acid): alanin, V: valine, T: threonine, P: prolin, CDS: coding sequences, NA: not applicable. Data sources: “National Center of Biotechnology Institute, SNP database (https://www.ncbi.nlm.nih.gov/snp/), Mutation Taster (https://www.genecascade.org/MutationTaster2021/#chrpos), SIFTonline tool (https://sift.bii.a-star.edu.sg/www/SIFT_dbSNP.html), PANTHER (https://www.pantherdb.org/tools/csnpScoreForm.jsp), VarSome (https://varsome.com/variant/hg38/) according to SwissModel 4nux 342–556. The VarSome 3D protein viewer for rs41323645 and rs4819555-related protein products was unavailable on the applicable model”. All databases were last accessed on 30 July 2023.

## Data Availability

All generated data in this study are included in the article and Supplement Materials.

## Supplementary Materials

The following supporting information can be downloaded at https://www.mdpi.com/article/10.3390/children11060657/s1, Table S1: Association of studied gene variants with allergies and aggravating factors in asthma. Table S2: *IL17RA* gene ontology.
