# Supplementary material for: Impact of Interleukin-17 Receptor A Gene Variants on Asthma Susceptibility and Clinical Manifestations in Children and Adolescents
Source: Children (Basel). 2024 May 28;11(6):657. doi: 10.3390/children11060657 (PMC11202101; doi:10.3390/children11060657)
Supplement: Supplementary file 1 [file children-11-00657-s001.zip › children-2992271-supplementary.pdf]

Table S1. Association of studied gene variants with allergies and aggravating factors in asthma.

**Risk factor**	**rs4819554*A carrier**		***p*-value**	**rs879577*C carrier**		***p*-value**	**rs41323645*A carrier**		***p*-value**	**rs4819555*C carrier**		***p*-value**
	**No**	Yes		**No**	**Yes**		**No**	**Yes**		**No**	**Yes**	
Sensitization to Allergens	5 (71.4%)	55 (61.8%)	0.71	3 (37.5%)	57 (64.8%)	0.15	16 (69.6%)	44 (60.3%)	0.47	9 (50%)	51 (65.4%)	0.28
Exposure to Animals	1 (14.3%)	21 (23.6%)	0.57	0 (0%)	22 (25%)	0.19	4 (17.4%)	18 (24.7%)	0.58	4 (22.2%)	18 (23.1%)	0.94
Food Allergies	3 (42.9%)	28 (31.5%)	0.68	3 (37.5%)	28 (31.8%)	0.71	8 (34.8%)	23 (31.5%)	0.80	4 (22.2%)	27 (34.6%)	0.41
Dust Allergy	1 (14.3%)	31 (34.8%)	0.42	2 (25%)	30 (34.1%)	0.71	11 (47.8%)	21 (28.8%)	0.13	5 (27.8%)	27 (34.6%)	0.78
Pollen Allergy	3 (42.9%)	22 (24.7%)	0.37	1 (12.5%)	24 (27.3%)	0.68	9 (39.1%)	16 (21.9%)	0.11	3 (16.7%)	22 (28.2%)	0.39
Exercise-Induced Asthma	4 (57.1%)	57 (64%)	0.70	5 (62.5%)	56 (63.6%)	0.95	15 (65.2%)	46 (63%)	0.85	8 (44.4%)	53 (67.9%)	0.10
Cold Air Intolerance	3 (42.9%)	44 (49.4%)	0.74	4 (50%)	43 (48.9%)	0.95	9 (39.1%)	38 (52.1%)	0.34	6 (33.3%)	41 (52.6%)	0.19
Aspirin Allergy	1 (14.3%)	21 (23.6%)	0.57	2 (25%)	20 (22.7%)	0.88	5 (21.7%)	17 (23.3%)	0.88	2 (11.1%)	20 (25.6%)	0.23
Allergic Conjunctivitis	1 (14.3%)	33 (37.1%)	0.42	1 (12.5%)	33 (37.5%)	0.25	12 (52.2%)	22 (30.1%)	0.08	2 (11.1%)	32 (41%)	0.03
Sinus or Ear Infections	3 (42.9%)	39 (43.8%)	0.96	3 (37.5%)	39 (44.3%)	0.71	14 (60.9%)	28 (38.4%)	0.09	5 (27.8%)	37 (47.4%)	0.19
Perfume Sensitivity	3 (42.9%)	41 (46.1%)	0.87	3 (37.5%)	41 (46.6%)	0.72	12 (52.2%)	32 (43.8%)	0.63	5 (27.8%)	39 (50%)	0.12
Respiratory Tract Infection	5 (71.4%)	55 (61.8%)	0.71	5 (62.5%)	55 (62.5%)	1.00	15 (65.2%)	45 (61.6%)	0.81	11 (61.1%)	49 (62.8%)	0.89
Emotional Stress Triggers	2 (28.6%)	34 (38.2%)	0.71	2 (25%)	34 (38.6%)	0.71	13 (56.5%)	23 (31.5%)	**0.047**	4 (22.2%)	32 (41%)	0.18
Exposure to Smoking	6 (85.7%)	54 (60.7%)	0.25	5 (62.5%)	55 (62.5%)	1.00	14 (60.9%)	46 (63%)	0.00	9 (50%)	51 (65.4%)	0.28
Anaphylaxis History	1 (14.3%)	19 (21.3%)	0.66	0 (0%)	20 (22.7%)	0.20	7 (30.4%)	13 (17.8%)	0.24	1 (5.6%)	19 (24.4%)	0.11
Urticaria (Hives) History	1 (14.3%)	27 (30.3%)	0.67	1 (12.5%)	27 (30.7%)	0.43	6 (26.1%)	22 (30.1%)	0.80	2 (11.1%)	26 (33.3%)	0.08

Dara are reported as frequency (percentage). Bold value indicates a statistical significance at *p*-value <0.05.

Table S2. *IL17RA* gene ontology

Accession	Term	Annotation source	Transcript IDs
**Cellular component**			
GO:0005576	extracellular region	UniProt	ENST00000319363 ENST00000612619
GO:0005886	plasma membrane	Ensembl	ENST00000612619 ENST00000319363
GO:0016020	membrane	UniProt	ENST00000694950 ENST00000612619 ENST00000319363
GO:0110165	cellular anatomical entity	UniProt	ENST00000612619 ENST00000319363
**Molecular functions**			
GO:0005102	signaling receptor binding	Ensembl	ENST00000319363
GO:0005515	protein binding	UniProt	ENST00000319363 ENST00000612619
GO:0030368	interleukin-17 receptor activity		ENST00000319363 ENST00000612619
**Biological process**			
GO:0002250	adaptive immune response	UniProt	ENST00000612619 ENST00000319363
GO:0002376	immune system process	UniProt	ENST00000612619 ENST00000319363
GO:0006954	inflammatory response	UniProt	ENST00000319363 ENST00000612619
GO:0007166	cell surface receptor signaling pathway	UniProt	ENST00000319363 ENST00000612619
GO:0009615	response to virus	UniProt	ENST00000319363 ENST00000612619

GO:0030163	protein catabolic process	Ensembl	ENST00000319363
GO:0032736	positive regulation of interleukin-13 production	Ensembl	ENST00000319363
GO:0032747	positive regulation of interleukin-23 production	BHF-UCL	ENST00000612619<br>ENST00000319363
GO:0032754	positive regulation of interleukin-5 production	Ensembl	ENST00000319363
GO:0032755	positive regulation of interleukin-6 production	UniProt	ENST00000612619<br>ENST00000319363
GO:0038173	interleukin-17A-mediated signaling pathway	UniProt	ENST00000612619<br>ENST00000319363
GO:0045087	innate immune response	UniProt	ENST00000319363<br>ENST00000612619
GO:0050729	positive regulation of inflammatory response	UniProt	ENST00000612619<br>ENST00000319363
GO:0050832	defense response to fungus	Ensembl	ENST00000319363
GO:0071621	granulocyte chemotaxis	Ensembl	ENST00000319363
GO:0072537	fibroblast activation	BHF-UCL	ENST00000319363<br>ENST00000612619
GO:0072538	T-helper 17 type immune response	Ensembl	ENST00000319363
GO:0097400	interleukin-17-mediated signaling pathway	UniProt	ENST00000612619<br>ENST00000319363
GO:1900017	positive regulation of cytokine production involved in inflammatory response	UniProt	ENST00000319363<br>ENST00000612619
GO:2000340	positive regulation of chemokine (C-X-C motif) ligand 1 production	UniProt	ENST00000612619<br>ENST00000319363

Data source: Ensembl release 110 - July 2023, (http://www.ensembl.org/).
